# Atmospheric Pressure Solvothermal Synthesis of Nanoscale SnO_2_ and Its Application in Microextrusion Printing of a Thick-Film Chemosensor Material for Effective Ethanol Detection

**DOI:** 10.3390/s22249800

**Published:** 2022-12-14

**Authors:** Nikita A. Fisenko, Ivan A. Solomatov, Nikolay P. Simonenko, Artem S. Mokrushin, Philipp Yu. Gorobtsov, Tatiana L. Simonenko, Ivan A. Volkov, Elizaveta P. Simonenko, Nikolay T. Kuznetsov

**Affiliations:** 1 Kurnakov Institute of General and Inorganic Chemistry of the Russian Academy of Sciences, 31 Leninsky pr., Moscow 119991, Russia; 2 Higher Chemical College of the Russian Academy of Sciences, D. Mendeleev University of Chemical Technology of Russia, 9 Miusskaya sq., Moscow 125047, Russia; 3 Basic Department of Inorganic Chemistry and Materials Science, National Research University “Higher School of Economics”, 20 Myasnsitskaya str., Moscow 101978, Russia; 4 Moscow Institute of Physics and Technology, National Research University, 9 Institutskiy per., Dolgoprudny 141701, Russia

**Keywords:** tin oxide, thick film, atmospheric pressure solvothermal synthesis, microextrusion printing, gas sensor, electrical conductivity, nanopowder, ink, ethanol, humidity

## Abstract

The atmospheric pressure solvothermal (APS) synthesis of nanocrystalline SnO_2_ (average size of coherent scattering regions (CSR)—7.5 ± 0.6 nm) using tin acetylacetonate as a precursor was studied. The resulting nanopowder was used as a functional ink component in microextrusion printing of a tin dioxide thick film on the surface of a Pt/Al_2_O_3_/Pt chip. Synchronous thermal analysis shows that the resulting semiproduct is transformed completely into tin dioxide nanopowder at 400 °C within 1 h. The SnO_2_ powder and the resulting film were shown to have a cassiterite-type structure according to X-ray diffraction analysis, and IR spectroscopy was used to establish the set of functional groups in the material composition. The microstructural features of the tin dioxide powder were analyzed using scanning (SEM) and transmission (TEM) electron microscopy: the average size of the oxide powder particles was 8.2 ± 0.7 nm. Various atomic force microscopy (AFM) techniques were employed to investigate the topography of the oxide film and to build maps of surface capacitance and potential distribution. The temperature dependence of the electrical conductivity of the printed SnO_2_ film was studied using impedance spectroscopy. The chemosensory properties of the formed material when detecting H_2_, CO, NH_3_, C_6_H_6_, C_3_H_6_O and C_2_H_5_OH, including at varying humidity, were also examined. It was demonstrated that the obtained SnO_2_ film has an increased sensitivity (the sensory response value was 1.4–63.5) and selectivity for detection of 4–100 ppm C_2_H_5_OH at an operating temperature of 200 °C.

## 1. Introduction

Tin dioxide has been attracting scientific attention all over the world for decades, both as an individual material [1,2,3,4] and as a component of more complex substances, especially transparent electrodes, including ITO [5,6], SnGaO [7], ZnSnO (ZTO) [8,9], SnO_2_:F (FTO) [10], Al-Sn-Zn-O (ATZO) [11], SnO_2_@N [12] and others. This material is a wide-bandgap semiconductor (E_g_ ~ 3.6 eV [1,3,13]) of n-type with a resistance of (1–3)·10^−3^ Ω·cm [14] and optical transmission in the visible light of 85% at 50–450 nm film thickness [1,14]. Among the functional characteristics, the gas sensitivity of tin dioxide is particularly prominent, and the spectrum of analyte gases is quite wide, including triethylamine (TEA, N(CH_2_CH_3_)_3_) [15], formaldehyde (HCHO), trichlorofluoromethane (CCl_3_F), methane (CH_4_), hydrogen sulfide (H_2_S) [16], benzene (C_6_H_6_), toluene (C_7_H_8_), ethanol (C_2_H_6_O) [15,17,18,19,20], heptafluorobutyronitrile (C_4_F_7_N) [21], nitrogen dioxide (NO_2_) [22,23], acetone ((CH_3_)_2_CO), isopropyl alcohol (IPA, C_3_H_8_O) [18], hydrogen (H_2_) [24], water vapor (H_2_O), carbon monoxide (CO) [25] and chloroform (CHCl_3_) [26]. Due to this fact, SnO_2_ is often used as the main component of chemoresistive gas sensors. It is also known that the microstructural and functional properties of metal oxides largely depend on the synthesis method used (sol-gel technology, chemical precipitation, glycol-citrate synthesis, hydro- or solvothermal method, gas-phase synthesis techniques, etc.). At the same time, the solvothermal method, due to the combination of simplicity and variability depending on the synthesis conditions, allows the formation of nanoscale tin dioxide with a particle size of 2–13 nm [22,24]. “Classically, this method is carried out in sealed reactors under elevated temperatures and pressures, which sometimes leads to technical complications and limitations, which can be avoided by using atmospheric pressure solvothermal (APS) synthesis” [27].

When manufacturing various devices, it is often necessary to form SnO_2_-based films. As a rule, such classical methods as dip-coating [28,29], spin-coating [30,31], physical vapor deposition (PVD) [32] and chemical vapor deposition (CVD) [26,33] are used for this purpose. These methods have some disadvantages, including limitations on the accuracy of dosing and targeting upon deposition of the material on the substrate surface, the risk of a gradient across the film thickness, high energy costs and precursor consumption, poor reproducibility, etc. Additive technologies, which enable the automated manufacturing of both bulk and planar materials, are attracting a considerable amount of attention today [34,35,36,37]. In this case, the reproducibility and targeting accuracy of the material application, as well as the speed of the process, increase significantly. One of the most promising and understudied methods of forming thick-film semiconductor materials, including chemosensory ones, is microextrusion printing [38,39,40,41,42,43,44,45], which is currently more frequently used for biomedical applications, in particular for printing organs and bones [46,47].

Thus, the purpose of this work was to study the atmospheric pressure solvothermal synthesis of nanoscale tin dioxide and its application in the formation of the corresponding thick film via microextrusion printing, as well as to study the microstructure and the electrophysical and chemosensitive properties of the obtained material. Particularly, it was necessary to evaluate the effectiveness of tin acetylacetonate as a precursor for the synthesis of tin dioxide with a developed surface that provides high chemosensory characteristics for the resulting oxide film.

## 2. Materials and Methods

### 2.1. Materials

A solution of tin acetylacetonate (0.2 mol/L) in acetylacetone (C_5_H_8_O_2_, 98%, Chimmed, Moscow, Russia), ethanol (C_2_H_5_OH, 96%, Chimmed, Moscow, Russia) and distilled water was used in this work without further purification. In order to measure chemosensory properties of the obtained SnO_2_ thick film, synthetic air (Zero Air Calibration Gas, oxygen volume fraction 20.9 ± 0.5%) was used, as well as calibration gas mixtures containing the following gases: 1% H_2_ (±0.015%), 200 ppm CO, 200 ppm NH_3_, 200 ppm C_6_H_6_, 200 ppm C_3_H_6_O (±5 ppm) and 200 ppm C_2_H_5_OH in air.

### 2.2. APS Synthesis of Nanosized SnO_2_

Tin dioxide nanopowder was obtained by APS method. In a typical experiment, a 6.5 mL solution of tin acetylacetonate in acetylacetone was added dropwise to a 120 mL mixture of ethanol and distilled water (C_2_H_5_OH volume fraction—30%) in a glass beaker under stirring, resulting in the formation of microemulsion and initiation of complex hydrolysis accompanied by the precipitation of solid phase particles. In order to intensify this process, the reaction system under stirring was heated up to 90 °C and kept at this temperature for 1 h. Separation of the precipitate from the mother liquor was carried out by stepwise centrifugation, after which the substance was washed twice with ethyl alcohol (dispergation was carried out using an ultrasonic bath) to remove acetylacetone formed as a by-product. Then, the precipitate was dried at 100 °C for 2 h in a drying oven in convection mode. At the next stage, the thermal behavior of the formed powder was studied using synchronous thermal analysis, and the conditions of additional heat treatment (400 °C, 1 h) required for complete decomposition of the semiproduct and formation of nanocrystalline SnO_2_ powder were determined.

### 2.3. Microextrusion Printing of SnO_2_ Thick Film

**Figure 1:**
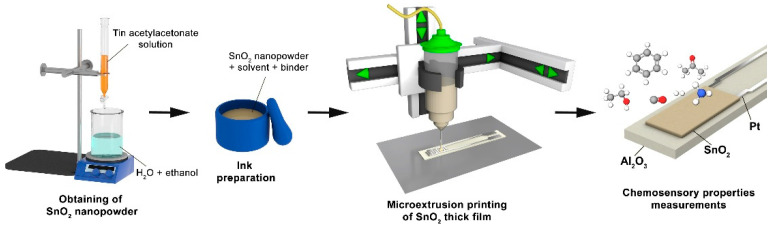
Scheme of SnO_2_ thick film formation.

The obtained tin dioxide nanopowder was further used to prepare a functional ink, which was a paste with a viscosity of about 0.5 Pa⋅s, in order to form a thick oxide film by microextrusion printing. For this purpose, the SnO_2_ powder was homogenized in α-terpineol in the presence of ethyl cellulose. Printing of the film on the surface of a specialized Pt/Al_2_O_3_/Pt-chip was performed using a three-coordinate positioning system and a pneumatic dispenser (system pressure 3.5 atm) equipped with a dosing device as well as G27-gauge needle (inner diameter 210 μm). The chip was an aluminum oxide substrate (Ra = 100 nm, geometric dimensions 4.1 × 25.5 × 0.6 mm) with platinum interdigitated electrodes (platinum layer thickness about 20 μm) on one side and a platinum meander microheater on the reverse side (Figure 1). The movement speed of the dispenser over the substrate surface was 0.5 mm/s, the pulse duration for paste dispensing was 0.8 s and the pulse interval was 0.5 s. The lateral dimensions of the formed oxide film were 5 × 3 mm. After printing, the film was dried at 40 °C for 24 h followed by a heat treatment at 400 °C (1 h) to remove residual solvent and to decompose the organic components. The thickness of the oxide layer corresponded to the thickness of the platinum interdigitated electrodes and was 20 µm.

### 2.4. Instrumentation

IR transmission spectra of powders as suspensions in vaseline oil placed between KBr glasses were recorded using an InfraLUM FT-08 FT-IR spectrometer (Lumex, Saint Petersburg, Russia) in the wavenumber range of 350–4000 cm^−1^.

The thermal behavior of the semiproduct was analyzed using a combined DSC/DTA/TGA analyzer SDT-Q600 (TA Instruments, New Castle, DE, USA) in Al_2_O_3_ crucibles in an air flow (250 mL/min) at a heating rate of 10 °/min in two modes (1—in the temperature range 25–1000 °C, sample mass 23 mg; 2—in the range 25–400 °C with holding at 400 °C for 1 h, sample mass 84 mg).

X-ray diffraction patterns of the semiproduct, SnO_2_ powder, and oxide film were recorded on a D8 Advance X-ray diffractometer (Bruker, Bremen, Germany, CuKα = 1.5418 Å, Ni-filter, E = 40 keV, I = 40 mA, integration time = 0.3 s/point, step = 0.02°) in the 2θ range of 10–80°.

The microstructure of the obtained SnO_2_ powder and the printed film was studied using a three-beam NVision 40 workstation (Carl Zeiss, Oberkochen, Germany) and a Solver Pro-M scanning probe microscope (NT-MDT, Zelenograd, Russia) using ETALON HA-HR probes with a W_2_C conductive coating (resonance frequency ~230 kHz, rounding radius <35 nm) in the modes of semicontact AFM, Kelvin-probe force microscopy (KPFM) and scanning capacitance microscopy (SCM).

The specific electrical conductivity of the SnO_2_ film was studied by impedance spectroscopy using a professional electrochemical workstation based on a P-45X potentiostat/galvanostat with an impedance measurement unit FRA-24M (Electrochemical Instruments, Chernogolovka, Russia). Impedance measurements were carried out in air in the temperature range of 50–350 °C in the frequency interval of 1 MHz–0.4 Hz. The temperature of the sample was maintained by applying a voltage (QJ 3003H power supply; Ningbo JiuYuan Electronic, Ningbo, China) to the platinum microheater on the back side of the chip, and was controlled by a thermal imager Testo 868 (Testo, Lenzkirch, Germany). The electrical resistance of the material was calculated using ZView Scribner Associates, Inc. (Southern Pines, NC, USA) software (Version3.3c).

The chemoresistive responses were obtained using a special laboratory setup, a detailed description of which can be found in our earlier papers [43,48]. To measure the signal at different humidity levels, a specialized setup with a barboter was used, and the humidity of the gas mixture was monitored using a digital flow-through hygrometer “Eksis IVTM-7 K” (Eksis, Zelenograd, Russia). The responses to hydrogen (H_2_), carbon monoxide (CO), ammonia (NH_3_), benzene (C_6_H_6_), ethanol (C_2_H_5_OH) and acetone (C_3_H_6_O) were measured using the appropriate calibration gas mixtures with air.

The response was calculated using the following ratio:

S = R_air_/R_g_, (1)

where R_g_ is the response at a given analytical gas concentration and R_air_ is the baseline response (synthetic air).

## 3. Results and Discussion

### 3.1. Thermal Behavior of the Semiproduct

**Figure 2:**
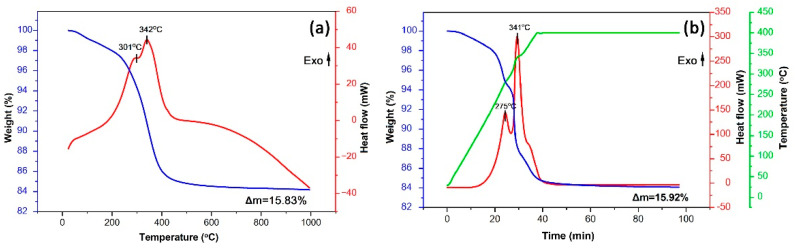
Thermal analysis results (weight—blue curve; heat flow—red curve; temperature—green curve) for the semiproduct in the temperature range of 25–1000 °C (**a**) and in the range of 25–400 °C with exposure to 400 °C for 1 h (**b**).

After drying at 100 °C, the thermal behavior of the obtained semiproduct was studied (Figure 2). In the first step, the analysis was carried out in a wide temperature range (25–1000 °C) (Figure 2a). As can be seen from the corresponding thermograms, when heating up to 250 °C, the mass loss of the powder is about 2.3%, which refers to the evaporation of residual solvents and sorbed atmospheric gases. Upon heating from 250 to 450 °C, the main stage of weight loss (11.7%), accompanied by two overlapping exothermic effects (with maxima at 301 and 342 °C) and related to oxidation of organic components contained in the powder, is observed. Further heating leads to slower mass loss (Δm is about 1.85% in the range 450–1000 °C). Thus, the total mass loss in the temperature range under consideration reached 15.83%, and the main processes occurred before 450 °C.

Next, in order to provide additional insights into the kinetics of the thermal transformation of the semiproduct, the dried powder was heated to 400 °C and exposed to that temperature for 1 h (Figure 2b). Powder mass was also increased to 84 mg (up from 23 mg) for this experiment in order to achieve more detailed thermograms. As can be seen, the steps of mass loss and the corresponding thermal effects became more distinct. Thus, in this case in the 250–400 °C interval, not two but three stages of mass loss accompanied by energy release are already observed. The maxima of the first two of these thermal effects, compared with the preliminary thermal analysis in a wider temperature range, shifts to the low-temperature region (down to 275 and 341 °C, respectively). In the 350–400 °C interval, an additional shoulder on the DSC curve is observed, along with a mass loss of about 3%. Thus, oxidation of the organic components contained in the semiproduct is a multistep process. During further exposure at 400 °C for 1 h, a significant slowdown of the powder mass loss is observed (Δm = 0.89%). As a result, it was determined that the value of the final mass loss (15.92%) in this regime of semiproduct heat treatment even exceeds the value that was recorded when heating to 1000 °C, i.e., the kinetics of the oxidation process plays a more important role than temperature increase. Using the results of synchronous thermal analysis, the mildest mode of heat treatment (400 °C, 1 h) was determined, which allows achieving the complete transformation of the semiproduct and provides preservation of a high dispersion state for the formed tin dioxide powder.

### 3.2. Crystal Structure, Spectral Properties and Microstructure of the Semiproduct and SnO_2_ Nanopowder

**Figure 3:**
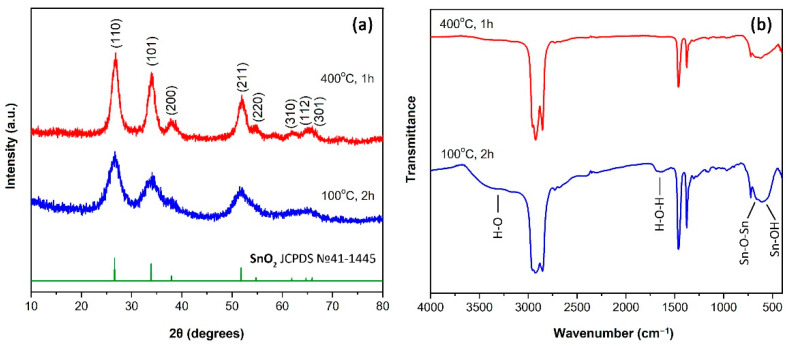
XRD patterns (**a**) and infrared spectra (**b**) of the semiproduct and SnO_2_ nanopowder obtained by additional heat treatment.

The crystalline structure of the semiproduct and the powder obtained as a result of additional heat treatment at 400 °C for one hour was studied by X-ray diffraction analysis (Figure 3a). Analysis of the corresponding diffractograms showed that the semiproduct is characterized by the crystal lattice of cassiterite (sp.gr *P*4_2_*/mnm*, JCPDS No. 41-1445), typical for tin dioxide. At the same time, no crystalline impurities were found in its composition.

As a result of additional heat treatment of the semiproduct, the crystal structure was preserved, but a decrease in the width and increase in the intensity of the reflexes can be observed, which indicates an increase in the average CRS size of X-rays. The average size of the CSR as well as the crystal lattice parameters for the powders under study were determined using full-profile analysis of the corresponding XRD patterns according to the Rietveld refinement method. Thus, for the semiproduct, the average size of the CSR was 4.1 ± 0.5 nm, and the parameters of the crystal lattice have the following values: a = b = 4.73 Å, c = 3.19 Å. Additional heat treatment at 400 °C for one hour led to an increase in the average size of the CSR to 7.5 ± 0.6 nm, as well as to some decrease in the crystal lattice parameters: a = b = 4.72 Å, c = 3.18 Å. In this case as well, no crystal impurities were revealed.

The set of functional groups in the obtained powders was studied by infrared spectroscopy (Figure 3b). Thus, unlike the SnO_2_ nanopowder obtained after additional heat treatment, the spectrum of the semiproduct shows absorption band in the range of 1550–1750 cm^−1^, as well as a broad absorption band in the region of 3000–3700 cm^−1^, which relate to the vibrations of OH-groups in the material composition. The presence of hydroxyl groups in this case is also confirmed by the presence of a band at about 600 cm^−1^ referring to the vibrations of the Sn-OH bond. In the case of both powders, in the wavenumber range of 450–800 cm^−1^ there is an absorption band characteristic of SnO_2_, relating to the vibrations of Sn-O-Sn bonds, which is in agreement with the results of XRD analysis. It should be noted that the IR spectroscopy results also indicate the absence of any impurities in the studied powders. The absorption bands observed in the spectra with maxima at 2900, 1400, 1300 and 750 cm^−1^ correspond to vibrations of functional groups of vaseline oil [49].

**Figure 4:**
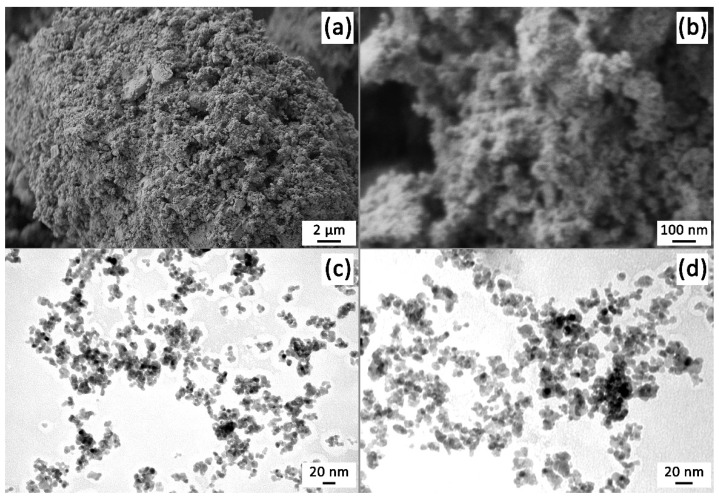
Microstructure of the obtained SnO_2_ nanopowder: ((**a**,**b**) SEM; (**c**,**d**) TEM).

The microstructure of the obtained SnO_2_ powder was studied by scanning (Figure 4a,b) and transmission electron microscopy (Figure 4c,d). As can be seen from the micrographs, the powder is characterized by a homogeneous microstructure, consisting of nanosized particles that are organized into globular agglomerates up to several tens of micrometers in size. According to SEM data, the average size of SnO_2_ particles is 21 ± 2 nm. In this case, no impurity formations differing in shape and size were detected in the composition of the powder. The TEM results made it possible to specify the size and shape of the oxide nanoparticles. Thus, it was determined that their shape was close to spherical, and their average size had a value of 8.2 ± 0.7 nm, which was comparable to the average CSR size determined by XRD analysis. Thus, it was found that the SnO_2_ powder obtained by the APS method is nanosized, has a homogeneous microstructure and does not contain any impurities.

### 3.3. Crystal Structure and Microstructure of Thick SnO_2_ Film

**Figure 5:**
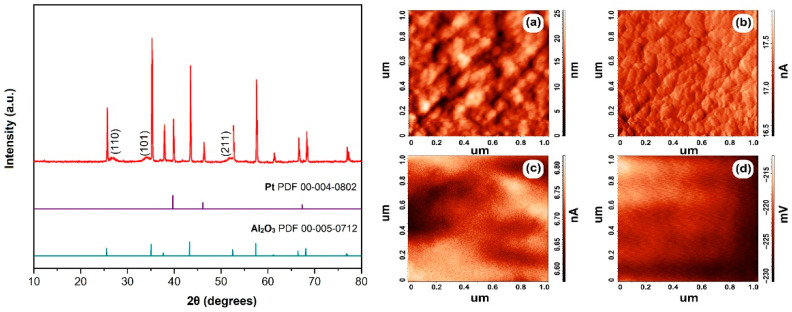
XRD pattern of the SnO_2_ film on the surface of a specialized Pt/Al_2_O_3_/Pt chip (**left**), as well as AFM results (**right**)—topography (**a**) and amplitude mismatch mode during topography imaging (**b**), capacity distribution map (**c**) and surface potential distribution (**d**).

The crystal structure of the tin dioxide thick film printed on the surface of a specialized Pt/Al_2_O_3_/Pt chip was studied by XRD analysis. As can be seen from the diffractogram (Figure 5, left), the main signals, which are intense narrow reflexes, refer to the substrate components (polycrystalline α-Al_2_O_3_ and platinum).

However, low-intensity reflexes of the applied SnO_2_ film are clearly visible in their background. At the same time, it was shown that after the oxide film had been printed on the substrate surface, the crystal lattice characteristic of tin(IV) oxide with a cassiterite tetragonal structure (sp.gr. *P*4_2_*/mnm*) was preserved. The analysis of the X-ray diffraction made it possible to estimate the average CSR size for the formed oxide film, which was 8.4 ± 0.7 nm. That is slightly higher compared to the nanopowder used for the preparation of functional inks. Nevertheless, the determined value of the average CSR size indicates that the material remains highly dispersed and that a nanocrystalline SnO_2_ film is obtained, while the slight increase in crystallite size can be explained by an additional heat treatment of the microextrusion-printed layer in order to remove the solvent and binder.

The microstructure as well as some local electrophysical properties of the formed tin dioxide film were studied by atomic force microscopy (Figure 5a–d). From the above scanning results (both topography and mismatch amplitude), it is seen that the film consists of round-shaped agglomerates with a diameter of about 50 nm, which are composed of smaller particles. For a scanning area of 100 μm^2^, the arithmetic average roughness was 166 nm and the mean square roughness was 212 nm. For a scan area of up to 1 μm^2^, the mean square roughness is 3.7 nm and the maximum height difference does not exceed 25 nm, indicating that the material is relatively even. From the SCM and KPFM results, it is clear that the distribution of electrophysical characteristics, such as charge carrier and surface potential distribution, is highly uniform. The value of the electronic work function of the SnO_2_ thick film under study was determined from the KPFM data, amounting to 5.048 eV, which is on the higher end of the values usually reported in the literature (4.7–5.2 eV [50,51,52]), which can be explained by the nanoscale state of the material or the conditions of coating heat treatment. According to published data, when tin dioxide nanoparticles are calcined, the energy of the conduction and valence band edges decrease, and the electronic work function value, therefore, increases [53].

**Figure 6:**
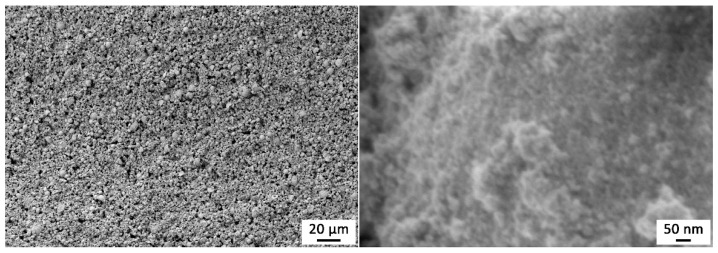
Microstructure of the printed SnO_2_ thick film.

The microstructure of the SnO_2_ film surface was also studied using SEM (Figure 6). As can be seen from the micrographs, the oxide film is porous (both nanopores and pores up to 5 µm in size are observed), characterized by a regular microstructure without defects in the form of cracks, fractures or delaminations. The presence of uniformly distributed oxide microspheres of 2–5 µm in size, which are formed at the first stage of APS synthesis due to the formation of microemulsion, can be seen in the structure of the material. Analysis of the images obtained at high magnification allowed us to determine the average particle size, which was 22 ± 2 nm. Thus, the application of the oxide film and its additional heat treatment had almost no effect on the size of the oxide particles of which it consists.

### 3.4. Electrical Conductivity of the Printed Oxide Film

**Figure 7:**
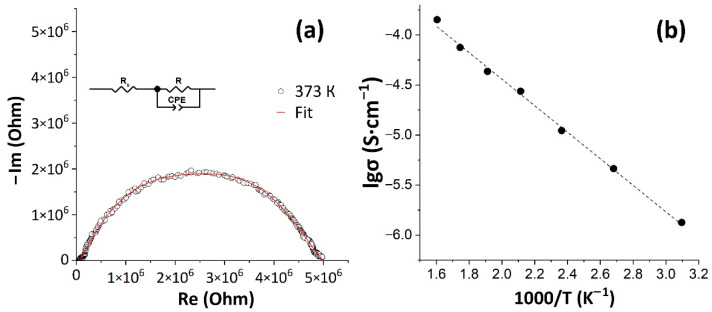
The impedance spectrum of a SnO_2_ thick film at a measurement temperature of 373 K (**a**) and temperature dependence of its total electrical conductivity (**b**) (the black markers indicate experimental values, and the dashed line is the result of linear approximation).

At the next stage the electrophysical properties of the SnO_2_ nanocrystalline film printed on the Pt/Al_2_O_3_/Pt chip surface were studied. Thus, using the impedance spectroscopy, frequency dependences of the complex impedance of the material in the temperature range of 50–350 °C within the frequency range of 1 MHz–0.4 Hz were obtained. Figure 7a shows an example of a typical hodograph obtained for the studied coating at a temperature of 100 °C. It can be seen that the hodograph has the form of a semicircle, slightly elongated along the abscissa axis, and can be described by an equivalent circuit including a resistance R (charge transfer resistance) and a constant phase element (CPE) connected in parallel. The series resistance (Rs) is the resistance provided by the wires and contacts that complete the circuit. A similar type of hodograph has also been observed previously in the literature for tin dioxide-based films [54]. The overall electrical conductivity of the samples was determined using resistance R values calculated from impedance spectra obtained in the temperature range of 50–350 °C.

As a result, it has been shown that the electrical conductivity of the coating linearly increased with temperature rising from 50 to 350 °C. Overall increase was by two orders of magnitude—from 1.23·10^−6^ to 1.22·10^−4^ S·cm^−1^ (Figure 7b). That is characteristic of tin dioxide-based semiconductor materials and is caused by ionization of defects (oxygen vacancies) which promotes the increase in concentration and mobility of charge carriers [55].

### 3.5. Chemosensory Properties of SnO_2_ Film

**Figure 8:**
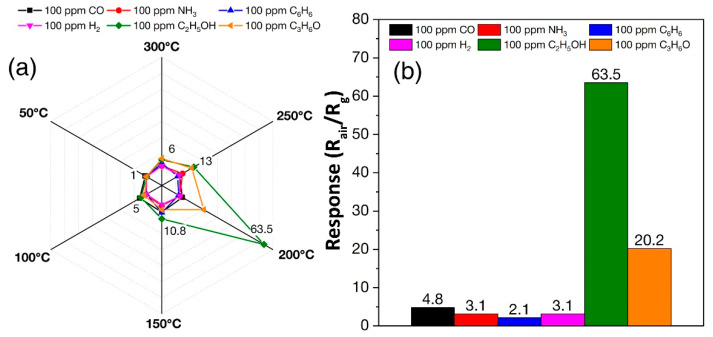
Selectivity diagrams showing the sensor responses of the SnO_2_ film to various analytes at operating temperatures of 50–300 °C (**a**) and at 200 °C (**b**).

The formed SnO_2_ thick film was also studied as a receptor component of a resistive gas sensor. At the first stage of the gas sensing study, the sensor responses to various analytes with concentrations of 100 ppm at different operating temperatures (50–300 °C) were registered in 50 °C increments. A selectivity diagram (Figure 8a) was constructed from the data obtained, demonstrating the dependence of the responses to the various analytes on the operating temperature. As can be seen, the highest response (10.8–63.5) was recorded for C_2_H_5_OH at operating temperatures of 150–250 °C with a maximum at 200 °C. The corresponding columnar selectivity diagram at 200 °C (Figure 8b) shows that the response to 100 ppm C_2_H_5_OH (S = 63.5) is significantly higher than that to 100 ppm H_2_, CO, NH_3_, C_6_H_6_ and C_3_H_6_O, indicating high selectivity of the studied SnO_2_ thick film in ethanol sensing.

**Figure 9:**
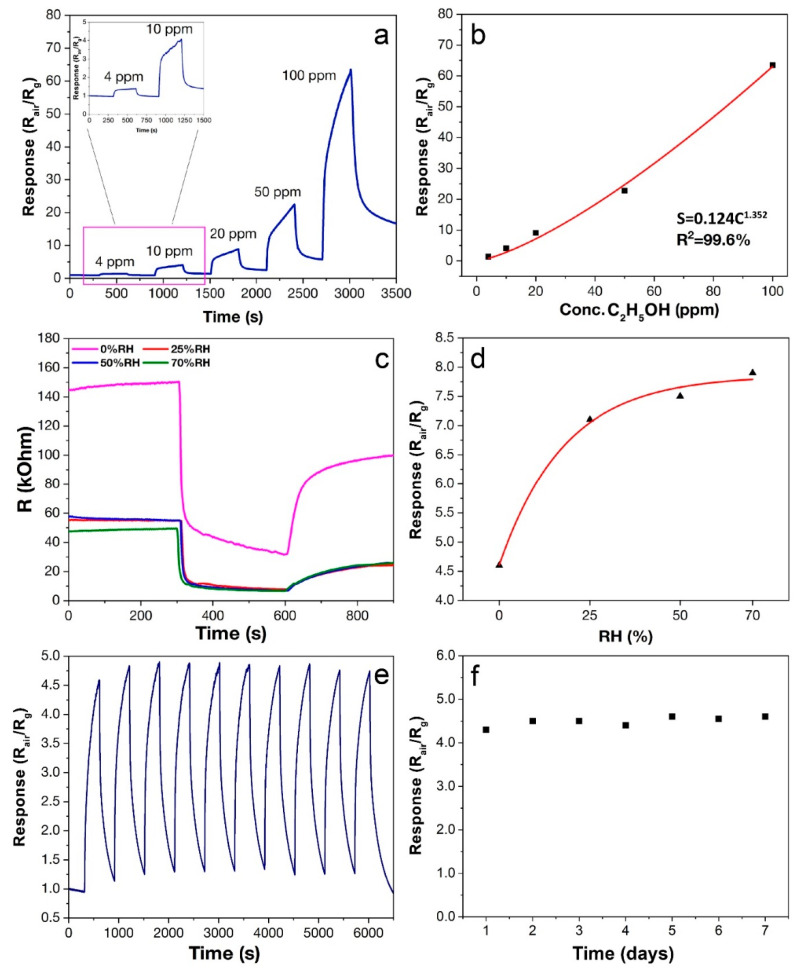
Dependence of the sensor response on the concentration (4–100 ppm) of C_2_H_5_OH (**a**,**b**), change of resistance upon detection of 10 ppm C_2_H_5_OH at 0–70% RH (**c**) and dependence of the response on 10 ppm C_2_H_5_OH at different humidity (**d**), signal reproducibility when detecting 10 ppm C_2_H_5_OH (**e**) and long-term stability of the SnO_2_ thick film (**f**). The operating temperature in all cases was 200 °C.

Data on the detection of various levels (4–100 ppm) of C_2_H_5_OH demonstrate (Figure 9a,b) that the response increases from 1.4 to 63.5 when the concentration increases from 4 to 100 ppm C_2_H_5_OH. The dependence of the response on concentration can be described by a power function that correlates with the Freundlich isotherm equation, widely used for chemoresistive gas sensors [56,57]:

S = *k*C*^a^*, (2)

where *k* and *a* are the proportionality and exponent constant and represent the adsorption capacity and adsorption intensification, respectively [58]. The parameter *a* directly indicates the sensitivity of the material to a particular analyte [56]. In our work, *a* = 1.352 (Figure 9b), which is a high enough value to quantify the concentration of ethanol in the gas mixture. Such a high ethanol sensitivity is related to the high dispersion state of the oxide nanopowder obtained by the atmospheric pressure solvothermal synthesis, and the high porosity of the SnO_2_ film ensured by the microextrusion printing method. It can also be associated with the rapid surface reactions between ethanol and ionsorbed oxygen at the specified operating temperature (200 °C).

The mechanism of ethanol detection by the SnO_2_ film can be described using the generally accepted model for n-type semiconductors. At elevated temperatures, sorbed oxygen ions (O^−^, O^2−^ and O_2_^−^) are formed on the surface of the material through the interaction of sorbed oxygen molecules with electrons from the SnO_2_ conduction band [59]. As a result, an electron depletion layer is formed on the semiconductor surface and the interaction with the analyte occurs with the participation of active oxygen ions (O^−^ particles prevail at 200 °C [60]).

During C_2_H_5_OH oxidation, various intermediate products are formed on the surface of SnO_2_ [61]: ethoxy groups (C_2_H_5_O^−^), acetaldehyde (CH_3_CHO), ethylene (C_2_H_4_), methane (CH_4_) and acetic acid (CH_3_COOH). However, the final products of the reaction are carbon dioxide and water [19,62]:

C_2_H_5_OH + 6O^−^ → 3H_2_O + 2CO_2_ + 6e^−^ (3)

The released electrons enter the conduction band of SnO_2_, resulting in a decrease in electrical resistance (Figure 9c) and the occurrence of a chemoresistive response. Analyzing the obtained signals when detecting ethanol (Figure 9a), it should be noted that their shape is sharp, which indicates a sufficiently long equilibrium establishment time. The ethanol oxidation process (Equation (3)) is a multistep process. As mentioned above, the resulting intermediate products directly affect the equilibrium establishment time, which is reflected both in the shape of the signal obtained and in the overall kinetics of the entire process.

In order to study the effect of humidity on the sensor response, the measurements on the detection of 10 ppm C_2_H_5_OH at 25, 50 and 70% RH were carried out (Figure 9c). As can be seen, in a humid environment there is a significant decrease in resistance both in the baseline (air) and upon ethanol detection. At increased humidity, water molecules react with the SnO_2_ surface to form hydroxyl groups [63,64]:

H_2_O + 2Sn_Sn_ +O^−^_ad_ ⟷ 2(Sn_Sn_^+^ − OH^−^) + e^−^ (4)

As a result of this reaction, additional electrons are generated, which enter the conduction band of SnO_2_, leading to a decrease in resistance, as was observed in our case. Hydroxyl groups on the SnO_2_ surface occupy active sorption centers, thereby reducing the number of sorbed analyte molecules, which leads in many cases to a decrease in the sensor-response value [19,65]. However, in our case, despite the decrease in resistance, the ethanol response value increased at elevated humidity (Figure 9d). Similar behavior has also been previously observed in a number of studies, although it is hardly typical [61,66,67]. There is no single explanation for this phenomenon, but it might be attributed to the formation of ethoxy groups (C_2_H_5_O^−^) on the SnO_2_ surface after the ethanol reaction with hydroxyl groups:

C_2_H_5_OH + HO^−^ → C_2_H_5_O^−^ + H_2_O (5)

The formed ethoxy groups can be further oxidized to CO_2_ and H_2_O [61]:

2C_2_H_5_O + 11O^−^ → 5H_2_O + 4CO_2_ + 11e^−^ (6)

The electrons released as a result of the reaction enter the conduction band of SnO_2_, which leads to a decrease in resistance. The reaction (6) is probably limited in a humid environment, which explains the increased response to ethanol. In the case of ethanol detection in a humid environment, this behavior is primarily associated with the intense surface reactions between ethanol, ionsorbed oxygen and hydroxyl groups at the specified operating temperature (200 °C). The reproducibility of the sensor signal when detecting 10 ppm C_2_H_5_OH (Figure 9e) and the long-term stability of the obtained thick SnO_2_ film (Figure 9f) were also evaluated in the study. As can be seen from the measurement results, the material shows high reproducibility and stability of chemosensor characteristics, which is extremely important for practical reasons.

Table 1 summarizes a literature analysis of ethanol-sensitive oxide materials used as the receptor layer for chemoresistive gas sensors since 2015. Different parameters were considered when evaluating the performance: operating temperature, analyte concentration, response value and relative humidity. As can be seen, the SnO_2_ thick film obtained in our study has a relatively low operating temperature for ethanol detection (200 °C), which is important with regard to the energy efficiency of gas sensors. Moreover, the obtained material is characterized by a low gas threshold starting from the concentration of 4 ppm, which is important for prompt impurity detection in the monitored atmosphere. In addition, it should be noted that this paper examined humidity effect (in a wide range) on the chemosensory properties of the obtained tin dioxide film, which is rarely found in the literature, although this parameter often has a fundamental influence on the functional characteristics of the material [68,69,70].

## 4. Conclusions

Atmospheric pressure solvothermal synthesis of nanocrystalline SnO_2_ (average CSR size—7.5 ± 0.6 nm; particle size—8.2 ± 0.7 nm), which was used as a component of functional inks for microextrusion printing of tin dioxide thick film on the surface of a specialized Pt/Al_2_O_3_/Pt chip, was studied with the use of tin acetylacetonate as a precursor. It was shown that the synthesized tin dioxide, as well as the film formed on its basis, have a cassiterite crystal structure. It was found that the average CSR size of the oxide film is slightly higher (8.4 ± 0.7 nm) compared to the nanopowder used for its fabrication, which is associated with additional heat treatment of the material. The nanoscale state of the SnO_2_ film was confirmed by scanning electron and atomic force microscopy and its local electrophysical properties were evaluated. In particular, the surface capacity and potential distribution maps were built. Using impedance spectroscopy, the temperature dependence of the specific electrical conductivity of the printed SnO_2_ film was determined—it was shown that the electrical conductivity increases by two orders of magnitude as the temperature rises from 50 to 350 °C. In addition, it was shown that the printed oxide film is a promising receptor component of resistive gas sensors for low concentrations of ethanol vapor. At the same time, the sensor response increases with humidity, which is not quite typical for such materials. Thus, our study confirmed that the use of tin acetylacetonate as a precursor makes it possible to obtain SnO_2_ powder with a developed surface, so that the resulting thick oxide film demonstrates high chemosensory characteristics in ethanol detection.

## Figures and Tables

**Table 1:** Characteristics of metal oxide chemoresistive gas sensors for ethanol.

Material	Temperature, °C	Concentration, ppm	Response (S)	S Calculation Formula	Relative Humidity, %	Reference
MoO_3_	250	100	19.8	R_air_/R_g_	0	[71]
Al_2_O_3_-ZnO	300	1	53	(ΔR/R_air_) × 100		[72]
ZnSnO	450	20–500	3.21–31.18	R_air_/R_g_		[73]
NiO	130	100	1.68		12	[74]
α-Fe_2_O_3_	300	100	29.64–37.57		~65	[75]
CuO	300–450	100	2.30–9.10		0	[76]
ZnO	RT/175 °C	2–100	22.9–59.6/90.5–321.7	(ΔR/R_air_) × 100	~55	[77]
In_2_O_3_	270	0.5–100	1.6–66	R_air_/R_g_	0	[78]
TiO_2_	350–450	50–150	1.8–6.5			[79]
V_2_O_5_	330	50–1000	1.19–9.09			[80]
SnO_2_	190	20–1000	35.6–120		50–70	[81]
SnO_2_	100–300	50	9–31		35 ± 5	[82]
SnO_2_	160–340	20–1000	21–50		0	[83]
SnO_2_	350	10–30	0.7–1	(ΔR/R_air_) × 100		[84]
SnO_2_	200–400	10–500	21.96–207.68	R_air_/R_g_		[85]
SnO_2_	150–300	2000	1.25–1.6	R_air_/R_g_		[86]
SnO_2_	230	200	24.9		30–90	[87]
SnO_2_	200	56–446	62–86	(ΔR/R_air_) × 100	0	[88]
SnO_2_	260	50–1000	274.5	R_air_/R_g_		[89]
SnO_2_	200–400	19.4–100	1–20			[90]
SnO_2_	150–300	100–500	40–157			[66]
SnO_2_	200	4–100	1.4–63.5		25–70	This work

## Data Availability

Not applicable.
